# Medial Prefrontal Transcranial Direct Current Stimulation Aimed to Improve Affective and Attentional Modulation of Pain in Chronic Low Back Pain Patients

**DOI:** 10.3390/jcm10040889

**Published:** 2021-02-22

**Authors:** Megan E. McPhee, Thomas Graven-Nielsen

**Affiliations:** Center for Neuroplasticity and Pain (CNAP), Department of Health Science and Technology, Aalborg University, 9220 Aalborg East, Denmark; mmp@hst.aau.dk

**Keywords:** high-definition transcranial direct current stimulation, conditioned pain modulation, low back pain, affect induction, distraction

## Abstract

Chronic low back pain (CLBP) is often without clear underlying pathology. Affective disturbance and dysfunctional pain mechanisms, commonly observed in populations with CLBP, have, therefore, been suggested as potential contributors to CLBP development and maintenance. However, little consensus exists on how these features interact and if they can be targeted using non-invasive brain stimulation. In this pilot trial, 12 participants completed two phases (Active or Sham) of high-definition transcranial direct current stimulation (HD-tDCS) to the medial prefrontal cortex, applied for 20 min on three consecutive days. Clinical pain ratings, questionnaires, and sensitivity to painful cuff pressure were completed at baseline, then 4 trials of conditioned pain modulation (CPM; alone, with distraction using a Flanker task, with positive affect induction, and with negative affect induction using an image slideshow) were performed prior to HD-tDCS on Day 1 and Day 4 (24 h post-HD-tDCS). At baseline, attentional and affective manipulations were effective in inducing the desired state (*p* < 0.001) but did not significantly change the magnitude of CPM-effect. Active HD-tDCS was unable to significantly alter the magnitude of the shift in valence and arousal due to affective manipulations, nor did it alter the magnitude of CPM under any basal, attentional, or affective manipulation trial significantly on Day 4 compared to sham. The CPM-effect was greater across all manipulations on Day 1 than Day 4 (*p* < 0.02) but also showed poor reliability across days. Future work is needed to expand upon these findings and better understand how and if HD-tDCS can be used to enhance attentional and affective effects on pain modulation.

## 1. Introduction

Patients with chronic low back pain (CLBP) often present without clear underlying pathology. However, these patients do commonly demonstrate affective disturbances [1] and impaired anti-nociceptive mechanisms [2,3]. These factors have been suggested as potential contributory mechanisms to CLBP development and/or maintenance [4], though there is little consensus on how these features interact and if they can be simultaneously targeted.

Anti-nociceptive mechanisms can be assessed in many ways and at various levels of the nervous system. One common psychophysical method to assess anti-nociception is to measure conditioned pain modulation (CPM), also known as the “pain-inhibits-pain” effect [5,6]. This measure gives an indication of net inhibition of a painful test stimulus, due to a heterotopic painful conditioning stimulus. Although initially thought of as a subcortical mechanism, CPM assessment in humans is also heavily influenced by cortical processes. Several experimental studies in healthy participants have shown distraction [7,8] and acute stress induction [9] to increase the magnitude of CPM, with suggestions of valence-specific effects on CPM also observed by the authors in prior work. This is consistent with effects of attentional and affective manipulation on individual painful stimuli in healthy participants (e.g., with distraction and positive affect reducing painfulness and negative affect often increasing painfulness [10,11]), though effects in patients with chronic painful conditions have been less clear. Among chronic pain populations, distraction may be less effective in reducing experimental pain perception [12,13], while affective manipulation may produce the expected effects [14], reduced effects [15], or even paradoxical effects on experimental pain perception and related cortical activity [16]. Nevertheless, it seems that attentional and affective manipulation may be able to enhance CPM efficiency, which may be further amenable to improvement by neuromodulation.

Non-invasive brain stimulation techniques have been used for various purposes, including managing chronic pain [17,18] and depression [19], and altering pain sensitivity [20]. Techniques have primarily targeted the motor cortex and dorsolateral prefrontal cortex, with some degree of preliminary success in reducing neuropathic pain [21], altering pain sensitivity, and acutely enhancing anti-nociceptive mechanisms [22]. However, the limited efficacy of existing paradigms in treating musculoskeletal pain conditions may be due to a lack of specificity in targeting disease-relevant pathways.

Recently, the medial prefrontal cortex (mPFC) was highlighted as a key locus for the interaction between pain, affective disturbance, and anti-nociception [23], due to its role in the encoding of negative affect and unpleasant sensations, and its projections to the periaqueductal gray (PAG) and thus descending noxious inhibitory pathways. In CLBP patients, affective disturbances together with observed alterations in connectivity between regions of the mPFC and PAG [24,25,26] indicate that the mPFC may be a relevant stimulation target for both affective pain modulation and anti-nociceptive mechanisms simultaneously.

The aim of this study was, therefore, to investigate whether (1) attentional and affective manipulation of CPM magnitude can be achieved in CLBP patients, and (2) if three consecutive days of active high-definition transcranial direct current stimulation (HD-tDCS) to the mPFC, versus sham HD-tDCS, was able to alter the attentional and affective manipulation of CPM. It was hypothesized (1) that attentional and affective manipulations that distract from pain and increase positive affect would enhance CPM magnitude, while negative affective manipulation would reduce CPM magnitude and (2) that active HD-tDCS would further enhance the effect of affective and attentional manipulations on CPM capacity.

## 2. Materials and Methods

### 2.1. Participants

CLBP patients were recruited via social media and local noticeboards. Eligibility was initially assessed via phone or email, then confirmed via anamnesis and physical examination at the first test session. Participants needed to be aged 18–60 years, speak and understand English, and report persistent pain (greater than 3/10 on average), that was present >3 days per week, posteriorly between the inferior border of the ribcage and lower gluteal fold, sufficient to limit daily activity for a period exceeding 3 months. Participants currently seeking active treatment, routinely taking analgesics or neuropsychotropic medications, with red flag symptoms, current or prior neurological, musculoskeletal, mental, or painful disorders were not eligible to participate. Participants also needed to pass the transcranial stimulation safety screen [27]. All eligible participants received written and verbal information about the study purpose and methods and provided written informed consent prior to inclusion.

An a priori sample size calculation was conducted in G*Power (v3.1.9.2, Heinrich-Heine University, Düsseldorf, Germany) for main outcome data (expected change in ramped CPM paradigm) from the main study this sample participated in, which is reported elsewhere (McPhee & Graven-Nielsen, unpublished). This suggested 12 participants would be sufficient to detect a significant (*p* < 0.05) moderate effect size (f > 0.25) with 80% power. As per this prior report, the trial was pre-registered on ClinicalTrials.gov (NCT03864822), approved by the local ethical committee (VN-20170034) and conducted in accordance with the Declaration of Helsinki. Data were collected at the Center for Neuroplasticity and Pain (CNAP), Aalborg University, in 2019, by a trained investigator (M.E.M.).

### 2.2. Study Protocol

**Figure 1:**
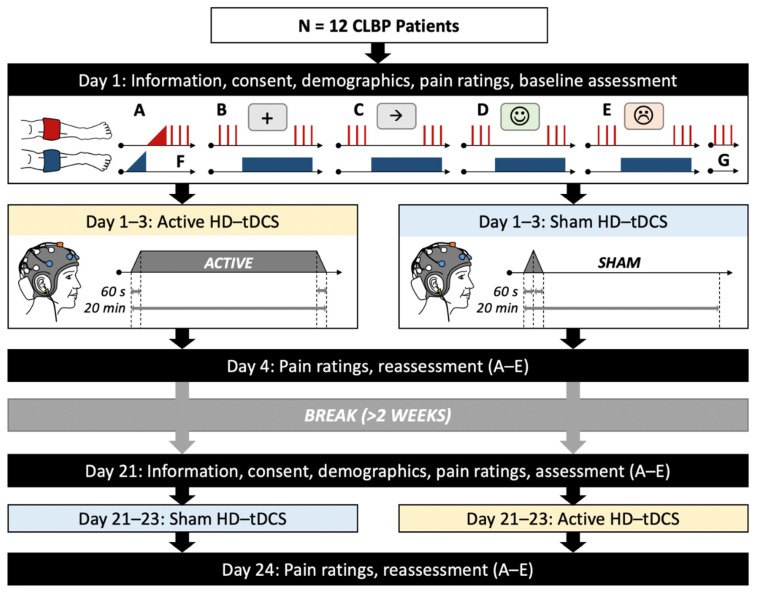
Schematic of chronic low back pain (CLBP) patient flow through study protocol. A: Cuff pain detection and tolerance threshold assessment, B: Conditioned pain modulation (CPM) only paradigm, C: CPM with distraction (Flanker task), D: CPM with positive affect induction, E: CPM with negative affect induction, F and G: control blocks of test stimuli. Note: order of B and C and D and E were randomized for each subject, as was assignment, to begin with active or sham high-definition transcranial direct current stimulation (HD-tDCS).

A cross-over placebo-controlled and double-blinded design was implemented. A computer-generated random number sequence was used to allocate participants to start with either the active or sham HD-tDCS condition. In each condition, participants attended 4 sessions on consecutive days where HD-tDCS was applied to the mPFC on Days 1–3. Prior to intervention on Day 1 and on Day 4, assessments were performed, including: (1) pain ratings, (2) basal pain sensitivity using cuff algometry, and (3) attentional and affective manipulations with simultaneous cuff paradigm to assess effects of attention and affective state on CPM. Each CPM manipulation trial was separated by at least 3 min with an interfering task to avoid carry-over effects [28,29,30]. Day 1–4 sessions were each separated by 24 h. After a break of at least 14 days, participants returned to complete the alternate condition (Figure 1).

### 2.3. Demographics and Pain Ratings

All participants reported their age, gender, body mass index (BMI), limb dominance and underwent a brief patient history and physical exam to confirm eligibility. A comprehensive assessment of pain history, sleep, mood, menstruation, physical activity, pain catastrophizing, anxiety, affect, disability, and pain features was also obtained from this population and will be reported elsewhere. Ratings of pain-related catastrophizing, on the Pain Catastrophizing Scale (PCS), baseline affective state, on the Positive and Negative Affective Schedule (PANAS), and state anxiety, on the Spielberger State and Trait Anxiety Inventory (STAI), will be reported here in correlations only. In each session, participants rated how much low back pain they were currently experiencing on both pain intensity and unpleasantness visual analog scale (VAS) anchored at 0 with “no pain/not unpleasant at all”, and 10 cm with “worst pain imaginable/most unpleasant sensation imaginable”.

### 2.4. High-Definition Transcranial Direct Current Stimulation

The HD-tDCS system consisted of a battery-powered multichannel neurostimulator (Starstim R32, Neuroelectrics, Barcelona, Spain) and neoprene EEG cap (NE056 Headcap, Neuroelectrics, Barcelona, Spain). Anodal direct current was delivered to the mPFC using five circular 1 cm diameter Ag/AgCl electrodes, with the anode positioned at Fz, four surrounding cathodes at F7, Fp1, Fp2, and F8, and a reference electrode on the right earlobe [31]. In both active and sham HD-tDCS phases, current amplitude increased over 60 s to the target intensity of 2 mA. In the active condition, the 2 mA was maintained for 18 min and then ramped off over 60 s (20 min total), whereas in the sham condition, the stimulator immediately ramped off again over 60 s and remained off for the subsequent 18 min.

### 2.5. Blinding of Participants and Experimenter

HD-tDCS paradigms were initially programmed in the HD-tDCS system by the experimenter (M.E.M.), then renamed and double-blinded using the HD-tDCS software’s inbuilt password-protected blinding feature by a colleague not involved in the study. Upon entering the study, participants were informed that they would receive two different stimulation types, with only one expected to have an effect but no further details about the different paradigms. Throughout the study, they were repeatedly told that stimulation would initially cause an itching, tingling, or warm sensation that would fade after the first few minutes in both conditions. Participants were debriefed on Day 4 of each condition to determine which condition they believed they had received and record side effects.

### 2.6. Cuff Pressure Algometry

A computerized cuff pressure algometry system (Nocitech and Aalborg University, Denmark) and two 10 cm-wide tourniquet pressure cuffs (VBM, Germany) were used to induce and measure deep tissue pain sensitivity. Participants were instructed to use both an electronic visual analog scale (eVAS; anchored at 0 cm: “no pain”, 10 cm: “worst pain imaginable”) and a verbal numeric rating scale (NRS; anchored at 0: “no pain”, 100: “worst pain imaginable”) to rate pain elicited by the cuff in different contexts.

Initially, a ramped cuff inflation at 1 kPa/s was applied to the non-dominant and then dominant leg. For each ramp, participants were asked to start sliding the eVAS dial upward when the pressure first became painful (cuff pain detection threshold, cPDT, eVAS = 1 cm) and press the stop button when they could not tolerate further increase in pressure pain (cuff pain tolerance threshold, cPTT). The final eVAS value before the stop button was pressed at cPTT was also extracted (eVAS@cPTT). This method offers a highly reliable user-independent estimation of pressure pain sensitivity [32].

### 2.7. Conditioned Pain Modulation Stimuli

During the 4 conditions with attentional and affective manipulation, a 2-min painful cuff conditioning stimulus was applied to the non-dominant leg at an intensity of 70% cPTT. The pain intensity elicited by this stimulus was rated on the eVAS by participants at the beginning and then adjusted at three time points (approximately 15, 30, and 45 s into conditioning) during each manipulation. As the eVAS records continuously, average ratings were extracted for the 0–15 s, 15–30 s, 30–45 s, and 45–75 s periods for analysis.

Test stimuli were applied 30 s prior to and 80 s into the conditioning stimulus of each manipulation. A test stimulus consisted of three 1 s stimuli separated by 10 s, for which the cuff inflated at 100 kPa/s to cPTT intensity on the dominant leg. Participants rated each inflation on the NRS and these were averaged for analysis. This method has been applied previously in healthy individuals and recurrent LBP patients showing similar effects to the traditional ramped CPM paradigm [33].

### 2.8. Manipulations

#### 2.8.1. Attentional Manipulation

The attentional task used was a version of the Flanker [34] programmed in E-Prime (Psychology Software Tools, Sharpsburg, PA, USA) to show a horizontal array of five arrows, in which participants should indicate the direction that the middle arrow was pointing (“1” for left and “3” for right on keypad). Arrows appeared randomly in one of four off-center locations on the screen with surrounding arrows pointing either in the same (congruent trials) or opposite (incongruent trials) directions to the middle arrow. Arrows were presented for 500 ms separated by 800 ms of a fixation cross, giving participants a total of 1200 ms to respond to each trial. Participants were initially familiarized with the task over 32 practice trials with unlimited response time and feedback on accuracy, then with a 1 min trial at proper speed without feedback.

During the CPM with distraction condition, a total of 48 trials were presented in the same manner as the practice phase, with the instruction to “rate pain now” from the cuff conditioning stimulation after every 12 trials. This task was synchronized with the beginning of the conditioning stimulus and ended immediately prior to reapplication of test stimuli.

During the CPM only condition, only a fixation cross was shown with instruction to “rate pain now” provided at matched time intervals. This presentation was similarly synchronized to conditioning stimulus application and ended immediately prior to reapplication of test stimuli.

CPM only and with distraction conditions were performed in random order but were consistent for each participant across the study. After both conditions, participants completed a brief mind-wandering questionnaire, with 6 questions regarding (1) their performance accuracy, how focused they were on, (2) the task, (3) sensations, (4) task-related thoughts, (5) unrelated thoughts, and (6) how much effort the task required. Each question was rated on a 7-point Likert scale from 1 (“never/not at all/none”) to 7 (“always/maximum”).

#### 2.8.2. Affective Manipulation

For affective manipulation, pictures were selected from the International Affective Picture System (IAPS, University of Florida, Gainesville, FL, USA [35]) to form four positive and negative image sets of 24 images (in 8 blocks of 3 contextually congruent images to improve valence shift [36]) each with similar content, valence, and arousal based on previously reported normative ratings [35]. Images were presented for 2000 ms followed by a 500 ms fixation cross, and after every 6 images, an instruction to “rate pain now” was shown.

As with the two attentional conditions, during both the CPM with positive affect and CPM with negative affect conditions, presentation of images was synchronized to start with the conditioning stimulus and ended immediately prior to reapplication of test stimuli. In these conditions, however, the final image in the affective manipulation slideshow was left on the screen until all pain ratings due to conditioning and test stimuli were complete, in an attempt to maintain affective state [37].

In each session, a different image set was used in an effort to avoid the effects of reduced novelty, and these were used in random order. Positive and negative affective manipulations were also performed in random order but were consistent for each participant throughout the study. After each affective manipulation, participants rated their affective valence with 1 (“most negative”) to 9 (“most positive”) and arousal with 1 (“very calm”) to 9 (“very aroused”) on the picture subscales of the Self-Assessment Manikin [35]. Participants were also asked, “What word would you use to describe how you felt while you watched the series of images?”.

All attentional and affective manipulations were completed sitting in a chair with back and arm support. Manipulations were presented on a 17″ monitor positioned approximately 50 cm in front of participants at eye-level.

### 2.9. Statistical Analysis

Cuff and pain rating data were checked for normality using Shapiro–Wilks and were analyzed using parametric or non-parametric approaches accordingly. Baseline clinical pain, cuff pain sensitivity (pain detection and tolerance thresholds), as well as control test stimuli and ratings of test and conditioning stimuli prior to attentional and affective manipulation were compared between HD-tDCS phases and days to assess for changes in basal sensitivity using repeated measures analysis of variance (RM-ANOVAs). To assess baseline effects of attentional and affective manipulation on CPM (Aim 1), test and conditioning stimuli were compared between manipulations (CPM only, CPM with distraction, Positive affect, or Negative affect) performed in the very first baseline session that patients attended regardless of HD-tDCS phase. Normalized changes in pain intensity scores for test stimuli (referred to as CPM magnitude, i.e., test stimuli NRS scores pre-manipulation subtracted from post-manipulation) and conditioning stimuli (referred to as normalized conditioning stimulus ratings, i.e., conditioning stimuli eVAS ratings for 0–15 s subtracted from 15–75 s) were then compared between HD-tDCS condition (Active, Sham), days (Day 1 to 4), and time (within session) throughout manipulation (where applicable) using RM-ANOVAs to assess the effect of HD-tDCS (Aim 2). Spearman’s Rho correlations were investigated between the effects of positive/negative affective manipulations on CPM magnitude and: (1) baseline affective state and anxiety (as per the PANAS and STAI data), and (2) evoked valence and arousal ratings. As well, Spearman’s Rho correlations were investigated between the effects of distraction on CPM magnitude and: (1) baseline pain and pain catastrophizing (as per PCS scores reported previously), and (2) the mind-wandering scale questions. Manipulation checks were performed to ensure affective induction was successful (i.e., that valence ratings were distinct and in the expected direction on the Self-Assessment Manikin), to confirm flanker effects (i.e., that incongruent trials were less accurate and required longer response time than congruent trials), and an additional check of reliability of these protocols was performed using two-way mixed intraclass correlation coefficients. Significance was accepted at *p* < 0.05 and data are presented as mean ± standard error of the mean (SEM) unless otherwise specified.

## 3. Results

### 3.1. Demographic Details and Pain Ratings

Twelve patients with CLBP (9 females, 3 males) were included in this trial and completed all four assessment sessions, though one participant stopped the active HD-tDCS after 10 min on Day 3 of that phase due to intolerable pulling sensations on the scalp. Participants were young (28.6 ± 5.9 years), with an average BMI (25.2 ± 4.4 kg/m^2^) slightly above normal. They reported having had CLBP for 5.3 ± 2.6 years with a current VAS pain intensity rating in the first session of 2.5 ± 2.0 cm and VAS pain unpleasantness of 3.1 ± 2.3 cm that did not change significantly between sessions (*p* > 0.12). All had sought medical or allied healthcare for their pain in the past, but none were currently undergoing treatment. Blinding was successfully maintained throughout the trial with similar rates of side effects (e.g., itching, tingling, and warm sensations during stimulation) between groups and guesses of assigned paradigm not significantly different from chance (50–67%), as will be reported elsewhere.

### 3.2. Cuff Pain Sensitivity

No differences were observed between HD-tDCS phases, days or legs for cPDT (grand mean: 21.5 ± 3.4 kPa; F < 4.1, *p* > 0.06, η^2^ < 0.28) or cPTT (grand mean: 45.0 ± 6.4 kPa; F < 3.8, *p* > 0.08, η^2^ < 0.26), suggesting test and conditioning stimulus intensities (which are based on these measures) did not differ significantly between sessions.

### 3.3. Baseline Effects of Attentional and Affective Manipulation

#### 3.3.1. Effects of Attentional and Affective Manipulation on Test Stimuli Ratings

In the very first baseline session, a main effect of time was observed (NRS-Pre: 32.9 ± 6.5, NRS-Post: 27.1 ± 5.4; F_1,11_ = 8.01, *p* < 0.02, η^2^ = 0.42), with a significant reduction in NRS ratings of test stimuli observed during conditioning (i.e., efficient CPM). However, no effects of or interactions with manipulation were observed (F < 0.35, *p* > 0.79, η^2^ < 0.04), suggesting that attentional and affective manipulation did not alter CPM effects.

#### 3.3.2. Effects of Attentional and Affective Modulation on Conditioning Stimuli Ratings

In the very first baseline session, a significant Manipulation × Time interaction was observed for eVAS ratings of conditioning stimuli (F_2.1,23.5_ = 3.76, *p* < 0.04, η^2^ = 0.25). On post-hoc testing, no differences were observed within timepoints between manipulations. Significant increases from initial (0–15 s) to all subsequent eVAS ratings (15–30 s, 30–45 s and 45–75 s) were observed in the CPM only (*p* < 0.03), CPM with distraction (*p* < 0.002), and CPM with negative affect (*p* < 0.05) conditions, whereas in the CPM with positive affect condition, increases were only seen from the first rating (0–15 s) to the second (15–30 s) eVAS rating (*p* < 0.002).

### 3.4. Pain Intensity of Control and Pre-Manipulation Test Stimuli

Ratings of control cuff test stimuli at the beginning and end of the test session revealed a main effect of Time, with end ratings (NRS = 30.7 ± 5.4) slightly lower than start ratings (NRS = 35.8 ± 5.5; F_1,11_ = 9.57, *p* < 0.01, η^2^ = 0.47). No differences were observed in start and end ratings between HD-tDCS phases or days, suggesting similar levels of pain induced by test stimuli between sessions. Pain intensity ratings of test stimuli prior to each attentional and affective manipulation showed no differences between HD-tDCS phases, days, or manipulations (NRS = 33.1 ± 21.5; F < 1.8, *p* > 0.21, η^2^ < 0.14), suggesting similar levels of pain was also induced by test stimuli throughout each session.

### 3.5. Effects of mPFC HD-tDCS on CPM Magnitude during Attentional and Affective Manipulations

**Figure 2:**
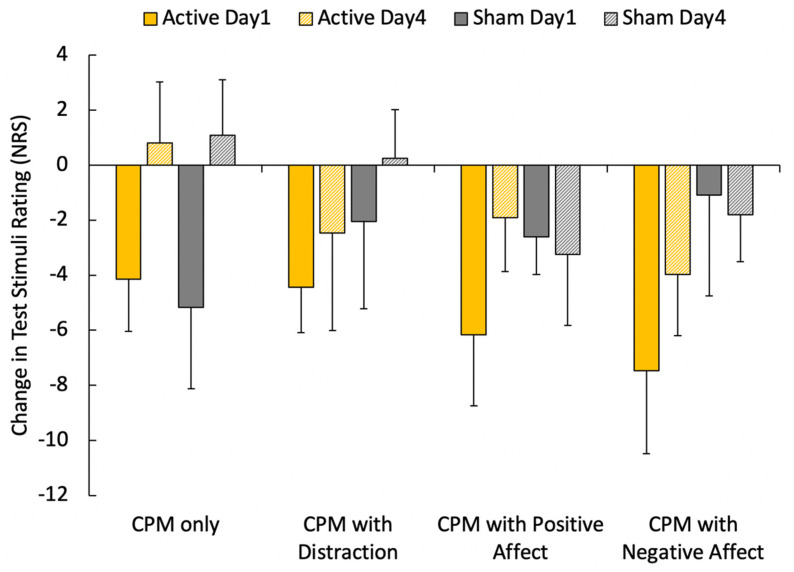
Pain modulatory effect shown as change in numerical rating scale (NRS) pain ratings (mean ± SEM) of test stimuli normalized prior to conditioning under the 4 conditions of conditioned pain modulation (CPM) without and with manipulations (during distraction from the flanker task, positive affect induction and negative affect induction using an image series). Negative values indicate the expected inhibitory response. No significant interactions were observed, only a main effect of Day with greater modulation on Day 1 than Day 4 (*p* < 0.02).

The magnitude of CPM during attentional and affective manipulations showed only a main effect of day (F_1,11_ = 7.36, *p* = 0.02, η^2^ = 0.40), with greater inhibition during manipulations generally demonstrated on Day 1 compared to Day 4 (Figure 2). No differences were noted between HD-tDCS phases or manipulations (F < 2.85, *p* > 0.12, η^2^ < 0.22).

### 3.6. Pain Intensity of Conditioning Stimulus Ratings Prior to Manipulation

Initial ratings of conditioning stimulus intensities were not different between HD-tDCS phases, days, or manipulations (eVAS: 2.8 ± 1.8 cm, F < 3.4, *p* > 0.09, η^2^ < 0.24), suggesting similar levels of pain initially evoked by conditioning stimuli between manipulations and sessions.

### 3.7. Effects of mPFC HD-tDCS on Normalized Conditioning Stimulus Ratings

**Figure 3:**
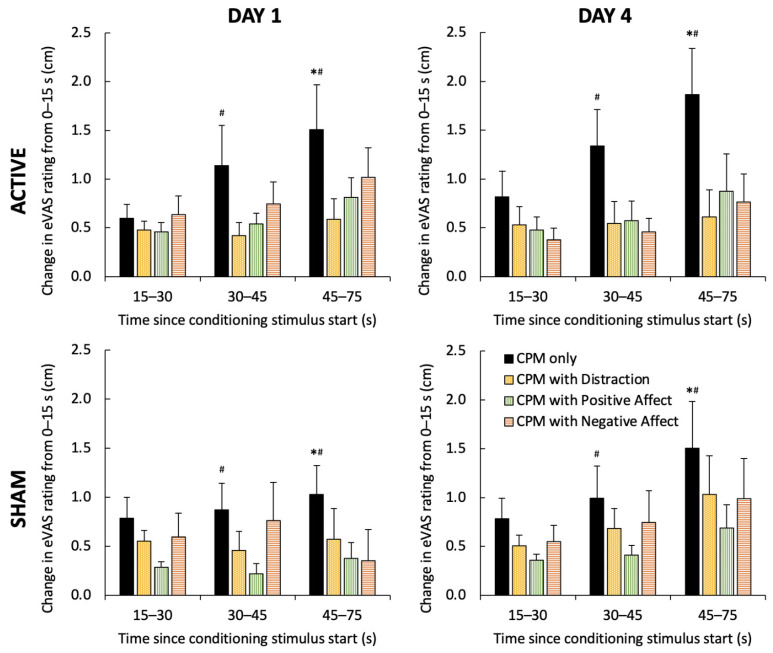
Increase in electronic visual analog scale (eVAS) conditioning stimulus pain intensity from first rating (normalized by subtraction) in the CPM only (black), CPM with distraction (yellow, spotted), CPM with positive affect (green, vertical), and CPM with negative affect (orange, horizontal) manipulations for each session (Active top row, Sham bottom row, Day 1 left column, and Day 4 right column). Significant increase in eVAS ratings compared to 15–30 s and 30–45 s (*, *p* < 0.02) and compared to all other manipulations (#, *p* < 0.01) indicated.

Changes in eVAS pain intensity ratings of conditioning stimuli throughout each manipulation revealed a Manipulation × Time interaction (F_6,66_ = 4.88, *p* < 0.001, η^2^ = 0.31), but no effects of HD-tDCS (F_1,11_ = 0.76, *p* > 0.40, η^2^ < 0.06) or Day (F_1,11_ = 1.07, *p* > 0.32, η^2^ < 0.09). On post-hoc testing, an increase in eVAS pain ratings was seen only during the cuff only trial (4th rating > 2nd and 3rd ratings, *p* = 0.011; Figure 3). Normalized eVAS ratings in the cuff only trial also showed greater increases than all other manipulation trials at the 3rd and 4th rating points (*p* < 0.01, Figure 3).

### 3.8. Manipulation Checks

Valence ratings on the Self-Assessment Mannikin during positive and negative affective manipulations showed a Day × Manipulation interaction (F_1,11_ = 8.51, *p* = 0.014, η^2^ = 0.44), whereby evoked valence ratings were higher in positive manipulations than negative on both days (Positive: 7.2 ± 1.4; Negative: 2.8 ± 1.2; *p* < 0.001). There was a tendency for higher valence ratings in the positive manipulation on Day 1 than Day 4, but this did not reach statistical significance (*p* = 0.071). No differences in arousal ratings were noted between HD-tDCS phases, days or manipulations (Positive: 4.1 ± 2.4; Negative: 4.4 ± 2.2; F < 2.5, *p* > 0.14). Overall, induced valence showed good to excellent reliability across sessions (Positive: ICC_3,k_ = 0.744 (0.514, 0.905), *p* < 0.001; Negative: ICC_3,k_ = 0.614 (0.339, 0.845), *p* < 0.001), while induced arousal only showed poor to moderate reliability across sessions (Positive: ICC_3,k_ = 0.385 (0.101, 0.711), *p* = 0.003; Negative: ICC_3,k_ = 0.556 (0.271, 0.815), *p* < 0.001). A more diverse array of words was used to describe the affective state induced by negative (*n* = 25) than positive (*n* = 19) manipulations, but in each valence condition there was one prominent word, namely, “happy” (19/48 trials) for positive manipulations and “sad” (14/48 trials) for negative manipulations. No significant differences were observed between HD-tDCS protocols or days for accuracy or reaction time during the Flanker task (*p* > 0.07), only that a Flanker effect was present for both accuracy and reaction time, i.e., with worse performance on incongruent trials (accuracy: 78.7 ± 16.1%; reaction time: 541.3 ± 69.1 ms) than congruent trials (accuracy: 97.6 ± 3.6%; reaction time: 489.0 ± 69.4 ms). Reaction time was also generally highly reliable between sessions (ICC_3,k_ = 0.714–0.726 (0.477, 0.897), *p* < 0.001).

### 3.9. Exploratory Correlations to Baseline State

**Figure 4:**
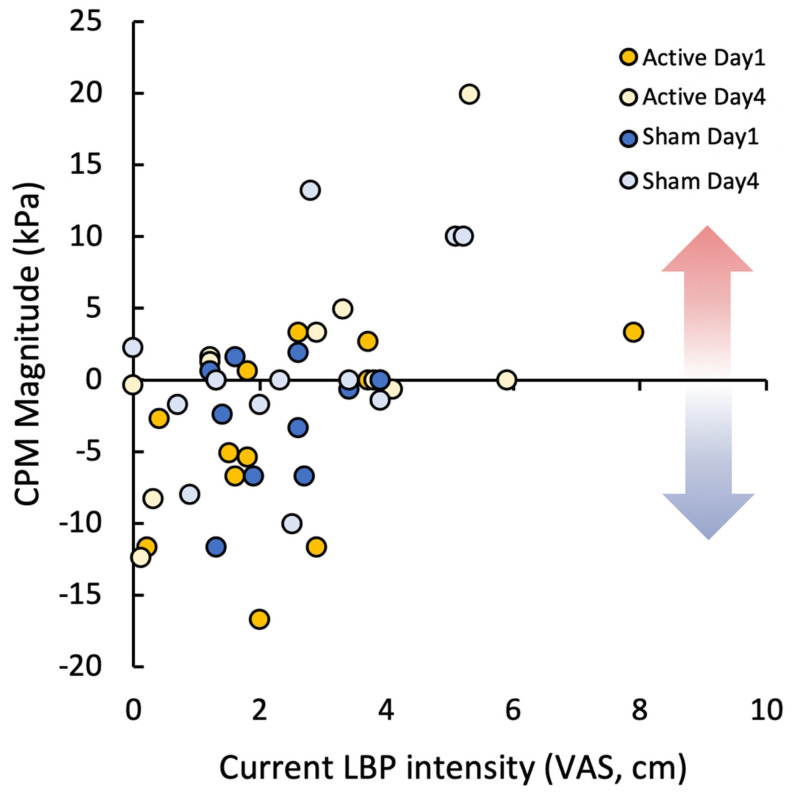
Scatter plot between CPM magnitude (cuff only) and current low back pain intensity scores on a visual analog scale for each session (Active Day 1, Active Day 4, Sham Day 1, Sham Day 4). Arrows indicate facilitation (red) and inhibition (blue) during CPM.

Significant correlations were observed between within-condition increases in eVAS pain ratings of conditioning stimuli during the positive manipulation and both baseline positive affect (PANAS, R_S_ = −0.516, *p* > 0.001) and state anxiety (STAI, R_S_ = 0.387, *p* = 0.007). Similar, though weaker, correlations were also observed for the other three manipulations with positive affect (PANAS, R_S_ < −0.329, *p* < 0.03) and for the CPM with distraction and negative manipulations with state anxiety (STAI, R_S_ > 0.318, *p* < 0.03). These correlations suggest that those with lower current positive affect and higher current anxiety show increased facilitation of pain perception during conditioning. No correlations were observed to PCS scores nor negative affect (PANAS) ratings. Current low back pain VAS intensity ratings correlated with CPM magnitude during the CPM only condition (R_S_ = 0.454, *p* = 0.001, Figure 4), indicating those with lower LBP intensity had better functioning CPM.

## 4. Discussion

In this trial, attentional and affective manipulations were unable to alter CPM magnitude in CLBP patients, and HD-tDCS of the mPFC was further unable to significantly enhance the effects of affective or attentional manipulation on CPM magnitude compared to sham HD-tDCS. Interestingly, pain modulation was generally better across all manipulations on Day 1 than Day 4 and generally did not show valence or task specificity. At baseline, pain ratings of conditioning stimuli were facilitated to some extent in all conditions, though not as consistently during the positive affect manipulation. Generally, these conditioning ratings also lacked valence-specific modulation and were instead reduced by all three manipulations compared to the cuff-only condition. The facilitation of tonic pain showed a correlation to baseline positive affect and state anxiety. Moreover, low back pain intensity was correlated with the amount of pain modulation in the CPM-only condition (i.e., CPM only was less efficient in patients with high pain intensity).

### 4.1. Efficacy of HD-tDCS in Altering Clinical Pain and Affective Responses

As reported previously, stimulation of the mPFC using HD-tDCS did not result in significant improvements in clinical pain or changes in affective measures. This study extends on that to suggest that HD-tDCS further does not have clear effects on affective or attentional manipulations, nor their impact on CPM magnitude. A number of factors related to the sample, namely, that they were young and presented with only mild pain and disability of relatively short duration (6 months to maximum 8 years), may explain why efficient CPM was observed despite evidence that this measure is typically impaired in CLBP patients [3]. This, in combination with the lack of affective disturbance in questionnaire data and intact ability to produce appropriate affective responses to manipulation, may further explain why improvements were not seen, as there was no clear impairment to target. It is also possible that significant improvements in the additive effect of affective and attentional manipulation could have been seen immediately following stimulation, but that it was simply not maintained to Day 4. However, as this was not assessed, this can only be speculated.

Interpretation of the present work is clearly complex, as these patients were expected to show both impaired CPM and impaired response to manipulations and have additionally shown non-response to HD-tDCS. This makes it difficult to conclusively state whether the lack of, e.g., valence specificity in pain modulation or analgesic effect of HD-tDCS, is due to characteristics of this specific sample or the methodology. However, the present methodology was based on prior literature and has been used successfully previously by the authors in other healthy and LBP populations. Future studies should, therefore, aim to better isolate effects of individual factors for comparison and consider non-inferential approaches to demonstrating potential evidence for specific null effects.

### 4.2. Time-Related Reduction in Pain Modulatory Capacity

It is unclear why less efficient pain modulation (under all attentional and affective conditions) was observed on Day 4 compared to baseline. As no differences were observed in psychological factors (PANAS, PCS, or STAI scores) between sessions or conditions, it is difficult to be certain, but this may be due to a reduction in contextual novelty. In line, the largest CPM magnitudes were observed in the absolute first session with, albeit non-significantly, less CPM observed in each subsequent session. Recently, there has been a tendency for studies to report a lack of significant improvement in CPM despite reduction or even resolution of pain [38,39], and in some cases temporal reduction in CPM with repeated assessments during the development of chronic pain [40,41]. Less is known about reductions in the efficacy of attentional and affective paradigms to modulate pain, as these are often performed in single session studies, but these manipulation effects are also likely to be impacted by stimulus and contextual novelty [42]. Although randomized stimulus presentation during the attention task and different image sets were used to maintain attentional and affective manipulation novelty, it was obviously necessary to use the same experimental setup, painful stimuli, experimenter, and laboratory in all sessions.

### 4.3. Lack of Valence Specificity in Pain Modulation

The International Affective Picture System has been frequently used as a method of inducing particular affective states, and this use of pictures has recently been highlighted as one of the best methods of experimentally inducing happiness and sadness [43]. In line, CLBP patients showed clear and differential expected shifts in valence following each affective manipulation. In the existing experimental literature, it is typical that positive affect induction will reduce pain perception where negative affect induction may even increase pain perception [44].

The effects of affective induction on pain perception have been attributed to both supraspinal mechanisms [45] and connections with descending inhibitory pathways [46], but interactions between experimental affective induction and psychophysical measurement of descending inhibitory function have scarcely been explored. As indicated, prior studies using affective manipulation have shown positive affect to reduce and negative affect manipulation to increase experimental pain perception [10]. In the present study, however, there was a distinct lack of valence specificity in pain modulation changes with CPM observed in all conditions on Day 1. Affect is believed to preferentially modulate pain unpleasantness [47], so it is possible that participants may have rated differently if they were asked to differentiate between pain intensity and unpleasantness; however, this was not possible here due to time constraints for ratings within the paradigm.

It is possible that valence-specific effects may simply have been too small to detect in the present sample, as prior work has also shown only minor effects of positive and negative image presentation on pain ratings, compared to pain-related image presentation [48]. Another explanation could be that, although CLBP patients reported appropriate shifts in valence, they may have rated what they thought the intended affective state was rather than their own present state. This is somewhat consistent with the fact significant correlations were observed between changes in pain perception during each manipulation and baseline positive affect and state anxiety levels, but not with valence ratings following manipulation. Prior work has shown a correlation between physiological measures, like zygomatic muscle activity, and subjective valence ratings [49], which could be a relevant addition to the present paradigm for future investigations to better evaluate this possibility. Finally, arousal may also be a critical factor in driving effects, as prior work reports significant modulation of pain by affective images compared to a neutral condition only when these images also had high arousal [50], and here most arousal ratings were low and did not differ between affective manipulations.

### 4.4. Lack of Additive Effect of Distraction on Pain Modulation

Beyond the lack of valence-specific effects, no differences were observed between attentional or affective manipulations, meaning distraction generally did not enhance pain modulation compared to CPM alone, in contrast to existing evidence [7,51,52]. This may be because test stimuli were re-assessed after task cessation, meaning the additional distracting effect was diminished. One prior study, using a stroop task, also concluded that distraction was not effective at enhancing CPM when these paradigms were sequentially applied, though this study also had difficulties in demonstrating significant CPM in healthy men at baseline [8].

### 4.5. Effects of Distraction on Facilitation of Conditioning Stimulus Pain

Distraction, whether due to the flanker task or to the images, clearly reduced facilitation of pain perception of the conditioning stimulus, consistent with prior literature on distraction and experimental pain perception [53,54]. However, in contrast to expectations based on the motivational priming hypothesis [55], there was no clear evidence of valence-specific effects, suggesting distraction may have been the primary driver in all conditions.

It should be noted that this reduction in conditioning pain due to the three manipulations may have negatively impacted CPM, as prior work has shown higher conditioning intensity to produce greater inhibitory effects [56]. This is interesting, given that objective pressure applied was identical between conditions and initial pain ratings were also not significantly different between attentional and affective manipulations. This point, therefore, warrants further investigation to understand whether perceived painfulness of conditioning throughout the stimulus or specifically when test stimuli are reapplied is more influential than objective stimulus intensity or initial painfulness.

### 4.6. Reliability of Affective Induction and Attentional Effects on Pain

Although there is a wealth of literature investigating the impact of attentional and affective manipulation on pain perception [10,11,48,52], and reverse effect of pain on cognitive performance and affect [57,58,59,60], there is little focus on the reliability of these interactive effects. Here, induced affective states, as rated on the Self-Assessment Mannikin, were highly reliable for valence and moderately reliable for arousal, despite the use of different image sets between sessions. The attentional performance was also highly reliable between sessions. Nevertheless, the resultant effects of these states on pain modulation were not at all reliable, even when only investigating baseline sessions without potential confounding effects of HD-tDCS response. Many studies have reported issues with CPM reliability generally [61,62,63], though the reliability of effects of additive paradigms remains largely unexplored. This is potentially problematic for longitudinal studies, or studies with multiple experimental sessions, looking at the interaction between affect, attention, and pain over time. In the present study, in line with temporal reductions in CPM magnitude generally within each phase, it is speculated that the lack of reliability may be due to: Reduced contextual novelty (as participants were exposed to the same series of tasks 4 times); Individual fluctuations in baseline affective state and attentiveness; Variation in engagement with the Flanker task; and nuanced affective responses to specific image sets (as captured by the wide variety of affective descriptors chosen). On this note, despite many studies using broad ratings of valence and arousal and classifying all affective experiences into the 6 basic human emotions [43], it is unclear how well these scales and discrete dimensions capture true affective responses.

### 4.7. Limitations

The small sample of patients in this study presented with mild pain and disability and showed little evidence of affective disturbance, which may explain the lack of efficacy of stimulation targeting these deficits in this group. In addition, affective manipulation reliably produced the desired affective state but did not have reliable effects on pain ratings. The sample was also primarily (75%) female, precluding sex comparisons, but given the well-established differences in e.g., chronic pain prevalence [64], the role of psychological factors [65], and CPM stability [66] between sexes, future studies should take this into consideration as some effects of affect manipulation and HD-tDCS may be sex-specific. Further, although this study was placebo-controlled, it lacked a healthy control group, which would have allowed for evaluation of how much the patients’ response, to the CPM paradigm, different manipulations, and HD-tDCS, deviated from normal. Future studies using more severely affected individuals are, therefore, warranted to better understand the impact of an attentional and affective manipulation on CPM responses and the additional effect of HD-tDCS techniques on this modulation. Additional investigation into the reproducibility and meaning of affective and attentional influences on CPM measurement is also needed.

## 5. Conclusions

This is the first study to investigate the additive effects of attentional and affective manipulation on CPM magnitude in CLBP patients and to attempt to enhance these interactions using mPFC HD-tDCS. The manipulations did not alter CPM magnitude at baseline and HD-tDCS was unable to significantly alter shift in valence and arousal due to affective manipulation, nor to impact effects of affective or attentional manipulation on CPM magnitude. General time-related reductions and poor reliability of affective and attentional effects on CPM and pain perception were observed, indicating the need for future work to clarify and improve on these aspects.

## Data Availability

The data presented in this study are available on request from the corresponding author.
